# Supplementary material for: Comprehensive Metabolomic Analysis Reveals Dynamic Metabolic Reprogramming in Hep3B Cells with Aflatoxin B1 Exposure
Source: Toxins (Basel). 2021 May 27;13(6):384. doi: 10.3390/toxins13060384 (PMC8229485; doi:10.3390/toxins13060384)
Supplement: Supplementary file 1 [file toxins-13-00384-s001.zip › toxins-1205213 SU Figures.pdf]

Supplementary Materials: Comprehensive Metabolomic Analysis Reveals Dynamic Metabolic Reprogramming in Hep3B Cells with Aflatoxin B1 Exposure

Shufeng Wang, Xin Yang, Feng Liu, Xinzheng Wang, Xuemin Zhang, Kun He and Hongxia Wang

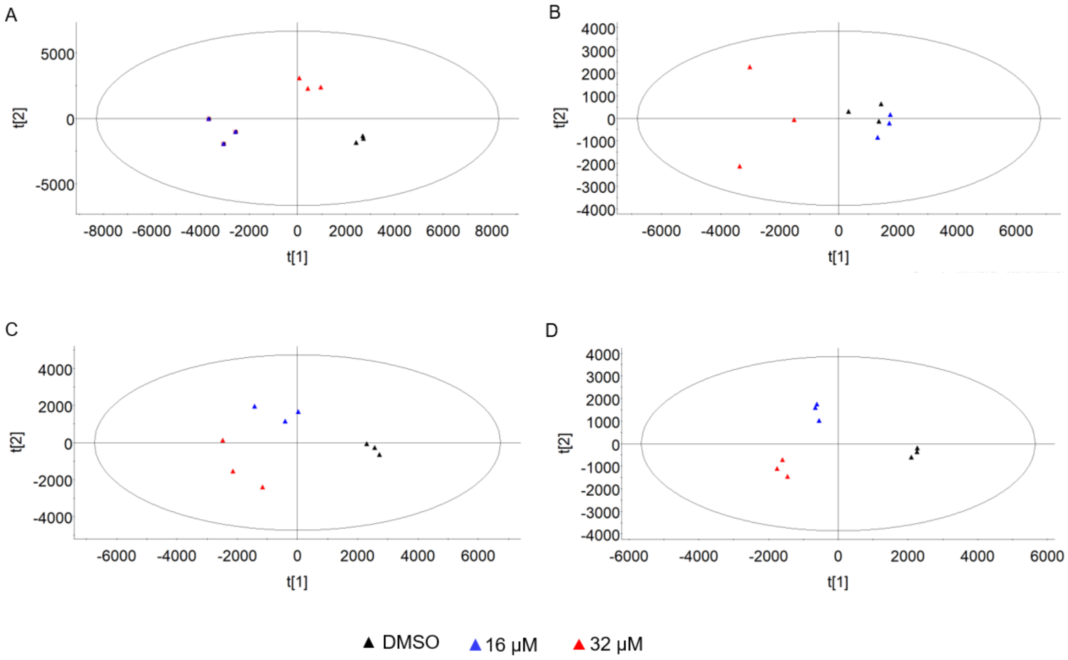

Figure S1. PLS-DA score plots based on Figure 3A–D, which were obtained by all metabolic features obtained from three biological replicates of AFB1 treated groups and control group by RPLC-POS (**A**), RPLC-NEG (**B**), HILIC-POS (**C**), and HILIC-NEG (**D**) modes, respectively. Samples with different treatments in (A–D) are illustrated at the bottom of the figure. DMSO: dimethyl sulfoxide; 16 μM: 16 μM AFB1; 32 μM: 32 μM AFB1.

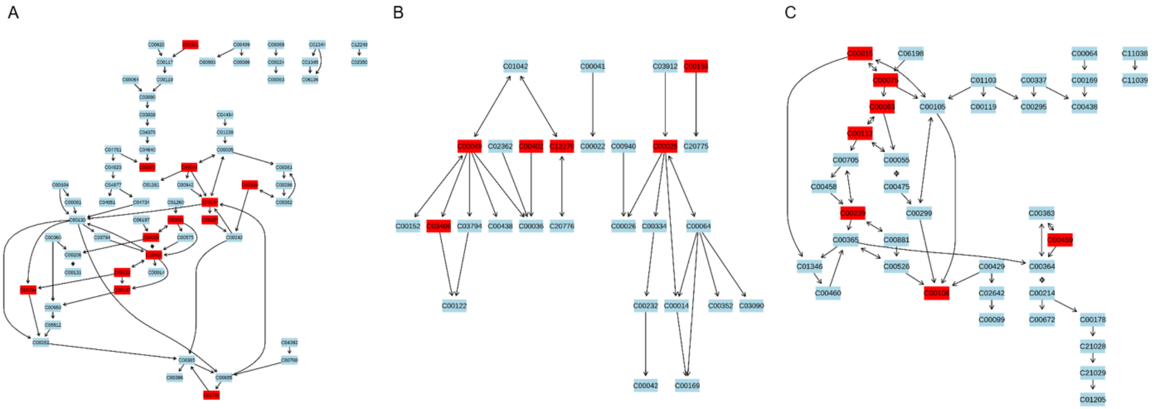

Figure S2. Venn diagram depicting overlap of metabolites in Hep3B cells differed significantly between each dose of AFB1treatment groups and the control group measured by four combined modes (RPLC-POS, RPLC-NEG, HILIC-POS, and HILIC-NEG).

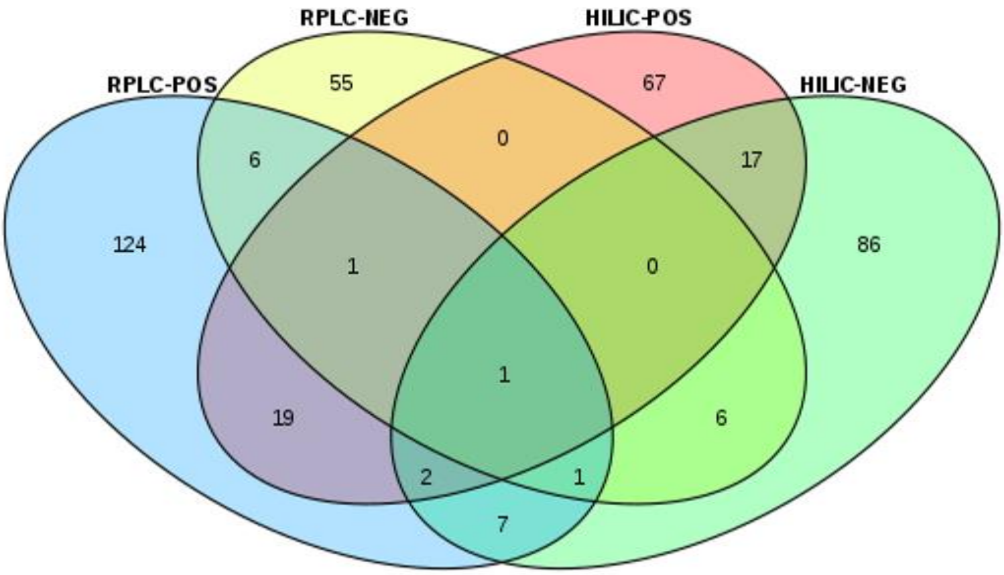

Figure S3. Constructions of the top three significantly altered metabolism pathways using the reference map by KEGG from 16 μM vs. DMSO comparison. CO represents the entry number of compound and the red ones were metabolites identified in this study: (**A**) Purine metabolism; (**B**) Alanine, aspartate and glutamate metabolism; (**C**) Pyrimidine metabolism.
